# Effect of Metformin or Chinese Herbal Formula in Patients with Type 2 Diabetes Mellitus and Hyperlipidemia: A Reassessment

**DOI:** 10.1128/mBio.01173-18

**Published:** 2018-07-24

**Authors:** Venu Gopal Jonnalagadda, Uppulapu Shravan Kumar, Shaik Afsar

**Affiliations:** a Department of Pharmacology and Toxicology, National Institute of Pharmaceutical Education and Research, C/O NETES Institute of Technology & Science, Mirza, Assam, India; b Department of Biotechnology, National Institute of Pharmaceutical Education and Research, C/O NETES Institute of Technology & Science, Mirza, Assam, India; c Department of Pharmacology, Narayana Pharmacy College, Chinthareddy Palem, Nellore, Andhra Pradesh, India; New York University

**Keywords:** gut microbiota, HbA1c, herbal, metformin, type 2 diabetes

## LETTER

We have read the article “Structural Alteration of Gut Microbiota during the Amelioration of Human Type 2 Diabetes with Hyperlipidemia by Metformin and a Traditional Chinese Herbal Formula: a Multicenter, Randomized, Open Label Clinical Trial” by X. Tong, et al. with great interest (1).

First, the authors did not mention the patient’s duration of diabetes mellitus and history of antidiabetic medication; this information is significant because antidiabetic agents could affect gut microbiota in different ways (1).

Second, in Table S2 in the supplemental material of their article, the value for HbA1c at week 0 was mentioned, but they did not present the value at the time of screening. Hence, we would like to know the mean value of HbA1c at the time of screening or before initiation of the washout period, which could show the exact value of HbA1c (1).

Third, after looking at the HbA1c values at week 0 in the metformin group, 8.13% ± 1.12%, and in the Chinese herbal group, 8.10% ± 1.23%, we believe that patients were diabetic (1). However, the patients had undergone a washout period for 4 weeks, so we would like to be aware of the rescue medications taken during this period.

We would be highly obliged for your kind consideration of the above-mentioned clarifications in your article to avoid discrepancy and highlight a clear idea of the amelioration of type 2 diabetes mellitus with hyperlipidemia by enriching beneficial bacteria using metformin or the Chinese herbal formula.
